# The impact of broad glutamine metabolism inhibition on the tumor microenvironment

**DOI:** 10.1016/j.gendis.2023.05.003

**Published:** 2023-06-20

**Authors:** Tianhe Li, Yun-Ta Chuang, Tian-Li Wang

**Affiliations:** Departments of Pathology, Oncology, and Gynecology and Obstetrics, The Sidney Kimmel Comprehensive Cancer Center, Johns Hopkins University School of Medicine, Baltimore, MD 21231, USA

Glutamine is an important α-amino acid that provides carbon and nitrogen sources for the biosynthesis of nucleotides, proteins, and lipids. It also generates metabolites that fuel the tricarboxylic acid cycle and maintain intracellular redox balances. Malignant cells have a heightened metabolic dependence on glutamine to support incessant tumor growth and mitigate tumor microenvironmental stresses including hypoxia, nutrient depletion, and oxidative stress. Somatic sequence mutations including MYC, TP53, Ras, NRF2/KEAP1, and PIK3CA, not only transform tumor cells but also alter their metabolic states, leading to an increased reliance on glutamine.^1^ Consequently, targeting the abnormal metabolism of tumor cells may be an effective strategy to starve cancer cells and induce anti-tumor effects. In this report, we highlight two recent papers that present significant advancements in the field of targeting glutamine metabolic pathways.^1^^,^^2^ Specifically, both research articles report the development of a promising glutamine antimetabolite and the potency of its analog in modulating the immune landscape. These findings hold great promise for further exploration and development of novel therapeutic agents that leverage the altered metabolism to treat different types of cancer.

There are three primary categories of drugs that potentially target the altered glutamine metabolism. They include glutaminase inhibitors, glutamine antimetabolites, and glutamine uptake inhibitors. Among them, 6-diazo-5-oxo-l-norleucine (DON), a glutamine antimetabolite, has demonstrated a potential for broad-spectrum anticancer therapy. DON is a nonselective inhibitor of glutamine-utilizing enzymes, and its structure is similar to that of glutamine. It acts as a competitive, irreversible inactivator of glutamine-utilizing enzymes, including glutaminase, a rate-limiting enzyme in the glutaminolysis pathway that plays critical roles in nucleotide and amino acid biosynthesis. Despite its promising anti-tumor efficacy in preclinical and clinical studies, concerns about its toxicity to healthy tissues, such as gastrointestinal tissue, have limited its clinical use.

To overcome this issue, a modified prodrug of DON, DRP-104, has recently been developed. DRP-104 carries two pro-moieties, an isopropyl ester, and an acetylated tryptophan, that allow it to be bioactive in tumors where proteases are abundant, and bio-inactive in gastrointestinal tissues by carboxylesterases. Furthermore, DRP-104 enhances the efficacy of anti-PD-1 immunotherapy through a CD8^+^ T cell-dependent mechanism, establishing immunologic memory that allows for immune clearance of tumor cells upon rechallenge. Lastly, DRP-104 is currently being investigated in a clinical trial (NCT04471415) in combination with anti-PD-L1 therapy for treatment in solid tumors.^1^

Preclinical *in vivo* studies using an analog of this class of glutamine antagonists JHU083 have demonstrated its differential metabolic effects on tumor cells and immune cells, including effector T cells^3^ and MDSCs^4^ resulting in a less immunosuppressive microenvironment and leading to the activation of cytotoxic effector T cells. Transcriptomic expression analysis of CD8^+^ tumor-infiltrating lymphocytes showed a decrease in hypoxia and oxidative stress, along with expression of markers associated with long-lived memory T cells.^3^

The primary metabolic effects of the glutamine antagonist on activated T cells are the preferential up-regulation of mitochondrial oxidative phosphorylation, mainly through glucose anaplerosis, to fuel the TCA cycle and maintain a balanced redox state. In contrast, for cancer cells, JHU083 suppresses glucose uptake and diminishes glucose metabolism in glycolysis and the TCA, leading to tumor regression.^3^

In a recent report, Huang et al evaluated the potential of JHU083 to enhance immunoprevention on an EGFR-mutated murine tumor mutation-driven lung cancer model.^2^ This type of lung cancer is poorly immunogenic and lacks infiltration of anti-cancer immune effector cells, resulting in a poor response to conventional anti-PD-1/PD-L1 treatment. The authors investigated the anti-cancer effects of adding JHU083 to an EGFR peptide vaccine (EVax) regimen. To better understand the specific impact on immune responses, the authors performed scRNA-seq on CD45^+^ populations.

Consistent with previous findings^3^, JHU083 treatment stimulated the effector-memory-like (EM-like) CD8^+^ T cells in mouse lung cancer, resulting from decreased activity in cell death pathways. Based on scRNA transcriptomic analysis, the authors further found an increase in a CD4^+^ subset, anti-tumor Th1 lymphocytes. Additionally, in the CD4^+^ subset, anti-tumor Th1 was increased, which was achieved through the up-regulation of the IL2-STAT5/mTORC1/Myc signaling pathways associated with Th1 cell differentiation, while pro-tumor Th17 and Tregs were decreased. The oncogenic signaling effectors, such as Stat3, p53, and Wnt, were inhibited in Tregs. In MDSCs, both granulocytic and monocytic subtypes were decreased under JHU083 treatment, potentially due to the decreased activities in apoptosis and autophagy. While the subsets of B cells also show a differential change by JHU083 treatment, the overall anti-tumor effect of these changes is less clear. Notably, NK cells and dendritic cells were not affected by the treatment.

The scRNA sequencing analysis conducted by Huang's research team provided new insight into the differential expression changes in cells within the tumor microenvironment following glutamine inhibition treatment. This approach has enabled a deeper exploration of the specific subsets of immune cells and their differential modulation by the drug.

In summary, the use of glutamine antimetabolites has shown significant anti-tumor efficacy both as a standalone therapy and in combination with other treatments, such as EVax. These compounds induce divergent metabolic responses in tumor microenvironment cells and trigger immunomodulatory effects (Fig. 1). However, current research has been limited to a small number of murine models with intact immunity. To gain a more comprehensive understanding of the systematic and local effects of this new class of glutamine antagonists, future research efforts should include metabolomic and single-cell RNA sequencing analyses on additional murine tumor models with immune phenotypes and disease presentations that simulate human diseases. This would greatly inform future therapeutic strategies targeting cancer metabolism.

**Figure 1:**
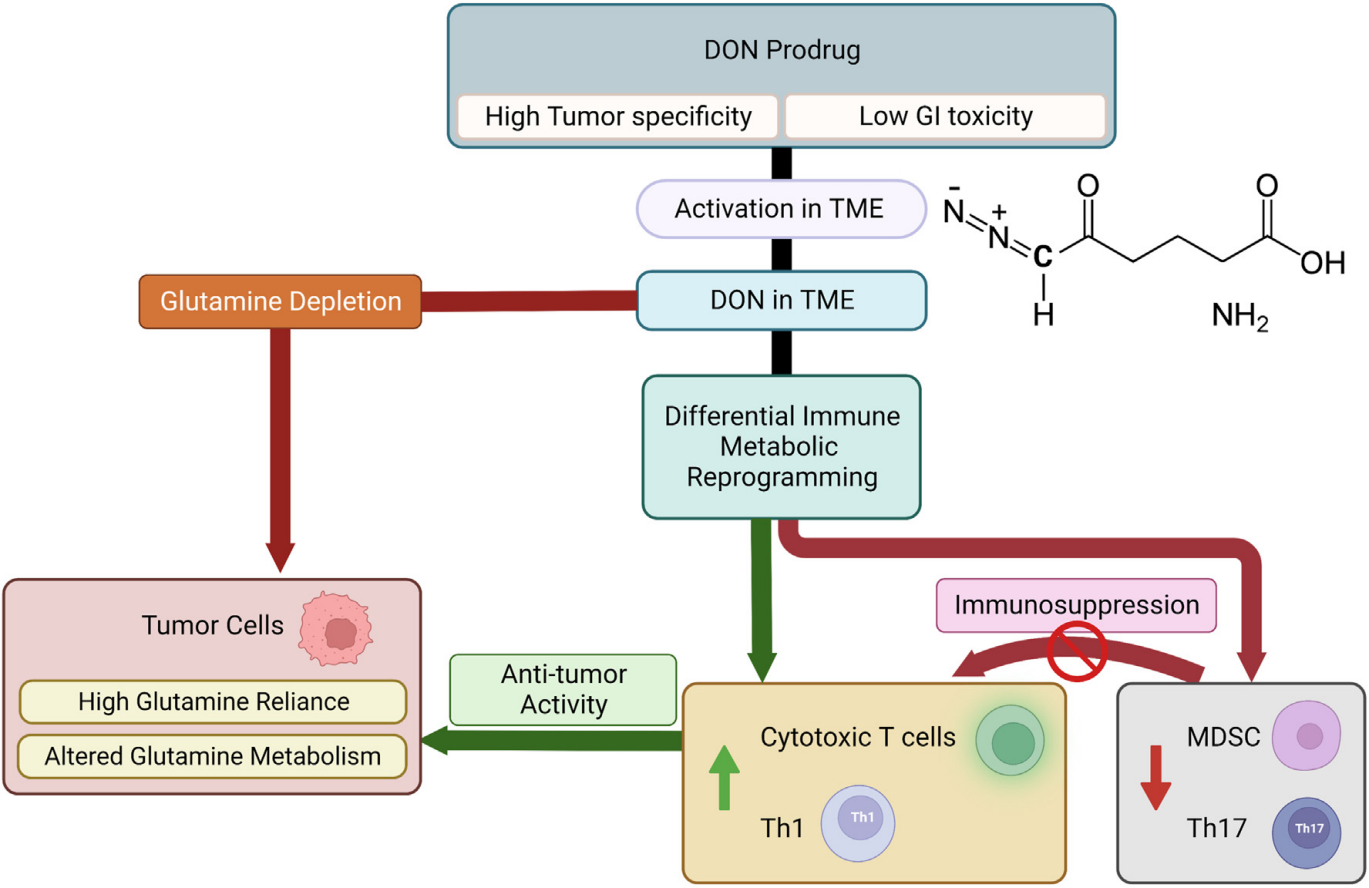
JHU083 and DRP-104 are prodrugs of glutamine metabolism inhibitor, DON, and they demonstrate selectivity towards tumor tissues through their preferential activation in the tumor microenvironment and inactivation in the gastrointestinal (GI) tissues. This differential activation of DON prodrugs into DON causes a distinct metabolic reprogramming of cell populations in the tumor microenvironment including the activation of anti-tumor cytotoxic T cells and Th1 cells, while simultaneously the suppression of the immunosuppressive MDSCs and Th17 cells. DON prodrugs also disrupt glutamine metabolism in tumor cells, which, unlike normal cells, are dependent on glutamine for their survival and proliferation. Consequently, the newly designed DON prodrugs exhibit potent anti-tumor effects through the dual mechanisms of metabolic inhibition and immunomodulation.

Although DRP-104 has been shown to suppress glutaminase, its *in vitro* metabolic and *in vivo* immunomodulatory effects differ significantly from glutaminase inhibitors, such as CB-839, which target only a single enzymatic pathway.^5^ Pre-clinical studies have shown potent anti-tumor efficacy when using JHU083 as a single agent, possibly due to the dependence of tumor cells on glutamine. DRP-104 is currently evaluated in phase 1 and phase 2a clinical trials for solid tumors, and the safety profile and patients' response to this promising metabolic drug will soon be revealed. Based on clinical findings, this class of drugs could potentially be used in combination with other standard-of-care cancer therapies. Overall, this research has significant potential for advancing cancer treatment, and further investigations are needed to determine its clinical benefits.

## Author contributions

All authors drafted and edited the manuscript. TL and TLW critically revised the manuscript and incorporated additional intellectual content. YC generated the graphical illustration.

## Conflict of interests

All authors declare that there is no potential conflict of interests.

## Funding

Supported by the Richard W. TeLinde Research Program, The Johns Hopkins University (USA).
